# Potential Mechanisms of Platelet Dysfunction and Bleeding in Acid Sphingomyelinase Deficiency

**DOI:** 10.3390/cells15151338

**Published:** 2026-07-26

**Authors:** Maksim Sysoev, Dmitri Solovyov, Aleksandr Shestopalov, Sergey Kutsev

**Affiliations:** 1 Research Centre for Medical Genetics, 1 Moskvorechye st., 115522 Moscow, Russia; soldmtrlx@gmail.com (D.S.); al-shest@yandex.ru (A.S.);; 2 Department of Biochemistry and Molecular Biology, Institute of Pharmacy and Medicinal Chemistry, Pirogov Russian National Research Medical University, 1 Ostrovityanova St., 117513 Moscow, Russia

**Keywords:** platelets, acid sphingomyelinase deficiency, acid sphingomyelinase, sphingomyelin, platelet dysfunction, hemostasis, lysosomes, lysosomal storage disease

## Abstract

**Highlights:**

**What are the main findings?**

- Models indicate that ASMD causes impaired platelet activation and changes in lipid metabolism and signaling.
- ASMD leads to disruption of coagulation factor system and endothelial function, further contributing to bleeding.

**What are the implications of the main findings?**

- The pathogenesis of hematological abnormalities in ASMD patients may involve direct effects of SMPD1 deficiency on platelet function and coagulation and not just be a byproduct of hypersplenism and platelet sequestration.

**Abstract:**

Acid sphingomyelinase deficiency (ASMD) is an autosomal recessive lysosomal storage disorder caused by mutations in the *SMPD1* gene, resulting in sphingomyelin accumulation. With a birth prevalence of 0.25–0.6 per 100,000, it is more prevalent in Ashkenazi Jewish and Middle Eastern populations. The disease features a clinical spectrum ranging from severe, early-onset neurodegeneration (infantile neurovisceral ASMD) to chronic, non-neurological visceral involvement (chronic visceral ASMD) and intermediate forms (chronic neurovisceral ASMD). The chronic visceral form is characterized by liver dysfunction, respiratory symptoms, and hepatosplenomegaly, which can lead to secondary thrombocytopenia. The majority of patients exhibit thrombocytopenia and/or mild bleeding manifestations, most commonly easy bruising and epistaxis, whereas clinically significant bleeding events, including gastrointestinal or variceal hemorrhage, occur less frequently. Systematic platelet-function studies in patients with ASMD are currently lacking. Evidence retrieved from biological models indicates that ASMD contributes to platelet dysfunction, including impaired secretion and thrombin generation, mediated by complex pathological mechanisms. These findings underscore the importance of hematological monitoring and further research in patients with this condition and could potentially offer new therapeutic possibilities.

## 1. Introduction

**Figure 1:**
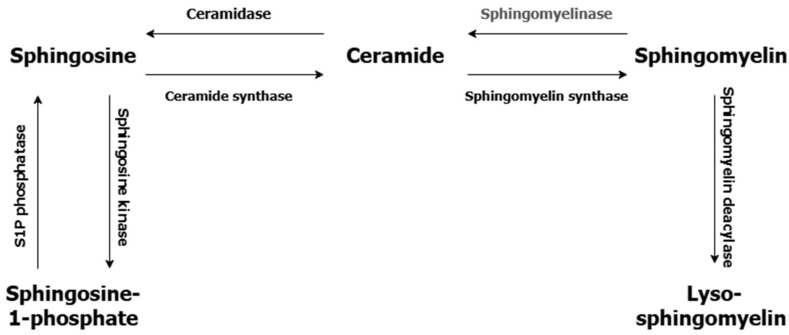
Diagram of the primary sphingomyelin metabolic pathways. ASMD is caused by a deficiency of acid sphingomyelinase, an enzyme that converts sphingomyelin into ceramide. Alternatively, sphingomyelin can be hydrolyzed by sphingomyelin deacylase into lyso-sphingomyelin, which serves as an important marker of ASMD. Ceramide may be further metabolized into sphingosine by ceramidase, and then into sphingosine-1-phosphate (S1P)—a biologically active sphingolipid that serves as a ligand for the S1P receptor family. Figure modified from Sysoev, M.; Solovyov, D.; Shestopalov, A.; Kutsev, S. Contradictory Effects on Hepatocytes in ASMD. *Int. J. Mol. Sci.* **2026**, *27*, 5070 [14], published by MDPI under a Creative Commons CC BY 4.0 license https://creativecommons.org/licenses/by/4.0, accessed on 20 June 2026.

Acid sphingomyelinase deficiency (ASMD), historically known as Niemann–Pick disease types A, B, and A/B, which correspond to infantile neurovisceral, chronic visceral, and chronic neurovisceral ASMD, is a rare genetic lysosomal storage disease caused by mutations in the *SMPD1* gene that result in the accumulation of sphingomyelin in lysosomes (Figure 1) [1,2,3]. ASMD has an average birth prevalence of 0.25–0.6 per 100,000 births. However, it is more prevalent in Ashkenazi Jewish and Middle Eastern populations, with prevalences of approximately 7.8 and 8.04 per 100,000 births, respectively [4,5,6]. The burden that the disease places on national healthcare organizations is significant [3,6,7,8,9,10,11,12,13].

Acid sphingomyelinase deficiency is divided into three forms based on the degree of neurological involvement: the infantile neurovisceral, chronic visceral, and chronic neurovisceral forms (Table 1) [15]. Patients with infantile neurovisceral ASMD have an early onset and a severe course of disease with early central nervous system damage; a lifespan longer than 2–3 years is uncommon for this form [16,17]. Patients with the chronic visceral form have no neurological involvement and usually exhibit a later age of onset and a significantly extended lifespan compared to patients with other forms of ASMD [18]. Chronic neurovisceral ASMD is a form with an intermediate degree of neurological involvement [15,17,19,20].

The main clinical signs of chronic visceral acid sphingomyelinase deficiency are hepatosplenomegaly, liver dysfunction, and respiratory symptoms [23]. Liver failure and respiratory failure are the most common causes of death in patients with ASMD [23,24]. Additionally, an increased bruising and bleeding risk is present in these patients, accompanied by thrombocytopenia [8,25]. In ASMD, one of the most dangerous and potentially life-threatening complications is variceal bleeding, which results from a combination of the high tendency for bleeding and liver failure [26,27,28,29].

### Article Selection Methodology

Articles for this review were identified through searches of the PubMed database focusing on acid sphingomyelinase, sphingolipid metabolism, platelet quantity, and platelet function. The literature search was conducted between 20 May and 29 June 2026, with the final PubMed search performed on 29 June 2026. Because of the limited literature directly addressing ASMD and platelet biology, multiple search strategies were employed using combinations of keywords, phrases, MeSH terms, Boolean operators, and field tags related to ASMD, sphingolipids, relevant enzymes, platelets, thrombocytopenia, coagulation, and platelet function. As a representative example, the PubMed query “(“Sphingomyelin Phosphodiesterase” [Mesh] OR (Sphingomyelin [OT] AND Phosphodiesterase [OT]) OR Sphingomyelinase [OT]) AND (“Blood Platelets” [Mesh] OR Thrombocyt* [OT] OR Platelet* [OT])” yielded 32 results, of which 7 directly addressed sphingolipid metabolism and platelet function and were included in this review. Additional studies were identified using other search strategies and by screening the reference lists of relevant publications. Preference was given to articles published within the previous 10 years, although older publications were included when appropriate. This review is narrative in nature; therefore, no formal systematic review methodology or PRISMA screening process was applied.

## 2. Hemostatic Abnormalities in ASMD

Hematological abnormalities and mild mucocutaneous bleeding manifestations are common in patients with ASMD. Approximately 63–64% of patients report at least one hematological or bleeding abnormality, most commonly thrombocytopenia, easy bruising, and epistaxis [30]. Clinically significant bleeding events, such as gastrointestinal or variceal hemorrhage, are less common but well documented; however, their prevalence remains unknown due to limited systematic reporting [26]. These bleeding tendencies may be explained by multiple phenomena, including reduced coagulation factor synthesis, local vascular factors, thrombocytopenia, and platelet function abnormalities. The latter seem to involve multiple mechanisms (Table 2). One of the key pathological mechanisms leading to thrombocytopenia in patients with ASMD involves platelet clearance from the circulation as a result of splenic sequestration driven by hepatosplenomegaly [19,31,32]. This is further supported by the fact that large spleen size is associated with low platelet levels and bleeding events in patients with ASMD [32,33]. However, based on existing data, the decrease in circulating platelet counts present in individuals with ASMD is usually not significant enough to cause the clinically relevant bleeding observed in these cases [34,35]. Sphingomyelin and cholesterol accumulation causes lipid composition shifts in cellular membranes, altering platelet membrane architecture and potentially resulting in defects of platelet adhesion and aggregation [31,36]. A potential disruption in calcium signaling may also compromise platelet function in ASMD patients, though this remains an untested hypothesis. To date, this mechanism has only been demonstrated in Niemann–Pick disease type C (NPC), a related disorder also resulting in cholesterol accumulation, which will be discussed later [31].

Olipudase alfa is a human recombinant acid sphingomyelinase that has gained prominence as a novel drug for enzyme replacement therapy of non-neurological (due to its inability to cross the blood–brain barrier) forms of ASMD [31,37,38]. Olipudase alfa therapy alleviates thrombocytopenia, presumably by reversing hypersplenism [10,39]. However, no studies have demonstrated that olipudase alfa normalizes qualitative platelet defects, such as impaired platelet activation and secretion, or abnormalities in thrombin generation, and this requires further investigation.

**Table 2:** Evidence supporting platelet abnormalities in ASMD.

Platelet Abnormality	Human Patients	Animal Models
Thrombocytopenia [19,31]	Present	Present
Reduced P-selectin expression [40]	Not studied	Present
Reduced ATP secretion [40]	Not studied	Present
Reduced thrombin generation	Not studied	Present
Impaired aggregation [40,41]	Not studied	Mixed evidence
Desialylation [42,43]	Not studied	Not studied
Ca^2+^ signaling defects [44]	Not studied, hypothesized from NPC1	Not studied, hypothesized from NPC1

### 2.1. Impairment of Platelet Activation in ASMD

The sphingolipid rheostat is regulated by compounds that double as platelet-activating agents, and, as such, influence various platelet functions [45,46,47,48,49]. Acid sphingomyelinase activity is present in human platelets, and is secreted extracellularly after in vitro platelet activation by thrombin, but not by ADP [50,51]. The increase in secreted ASMase is accompanied by a loss of intracellular lysosomal ASMase, and is reproduced by phorbol ester and a calcium ionophore, implying that protein kinase C (PKC) and intracellular calcium play a role [52]. Following thrombin activation of platelets, the ceramide amount is not significantly increased [53], while the sphingosine amount is elevated, which suggests high ceramidase activity in the cell [50]. Sphingomyelin-derived ceramide is a source of sphingosine-1-phosphate in platelets [54]. The exact function of secretory ASMase—whether platelet-derived, platelet-modulating, or both—remains poorly understood, but the increase in ceramide launches energy metabolism changes in platelets at rest [55] and the relocalization of enzymes involved in signaling pathways [56].

Acid sphingomyelinase plays a key role in sustaining platelet activation, especially at lower concentrations of the activator. Collagen-related peptide (CRP)-induced or low-level thrombin-induced ATP release and P-selectin expression are significantly lower in platelets from *SMPD1* knockout mice compared to platelets from wild-type mice. Phosphatidylserine expression on the platelet membrane after activation with thrombin and subsequent thrombin generation are notably lower in *SMPD1* knockout platelets than in wild-type platelets. Platelet integrin αIIbβ3 activation and aggregation, as well as activation-dependent calcium flux from the dense tubular system, do not differ between *SMPD1* knockout platelets and wild-type platelets. In vitro thrombus formation at a shear rate of 1700 s^−1^ and in vivo thrombus formation after FeCl_3_-induced injury are remarkably lower in *SMPD1* knockout mice, whereas bleeding time does not differ significantly. Platelets from *SMPD1* knockout mice show remarkably lower ceramide formation post-activation; furthermore, exogenously added ceramide restores the impaired platelet secretion and thrombus formation caused by ASMase deficiency. Adding bacterial sphingomyelinase to wild-type platelet suspension increases, whereas treatment with functional inhibitors of acid sphingomyelinase (FIASMAs) [57], such as amitriptyline or fluoxetine, lowers, activation-dependent platelet degranulation, phosphatidylserine exposure, and thrombus formation. The impaired thrombus formation and degranulation of platelets in *SMPD1* knockout mice are reversed by the addition of exogenous bacterial sphingomyelinase [40]. Historically, it has been challenging to determine exactly whether the role of ASMase in thrombus formation or activation of platelets is a direct effect of ASMase on platelet degranulation, its effect on increasing sphingomyelin, or potentially the accumulation of a secondary metabolite [58]. Recent data suggest that the situation is more complex, and there are multiple mechanisms in ASMD by which hemostasis is impaired.

The addition of lyso-sphingomyelin (lyso-SM), a deacylated form of sphingomyelin that is elevated in the plasma of ASMD patients [59], inhibits ATP release, P-selectin expression, α_IIb_β_3_ integrin activation, platelet aggregation, and thrombus formation in CRP-activated murine wild-type platelets. Consistent with previous findings, *SMPD1* knockout murine platelets exhibit diminished ATP release, P-selectin expression, and thrombus formation, while maintaining aggregation and α_IIb_β_3_ activation levels comparable to those of wild-type platelets [40,41]. A new finding is that lyso-SM does not alter ATP release, P-selectin expression, or thrombus formation in *SMPD1* knockout murine platelets; however, it does decrease their α_IIb_β_3_ integrin activation and platelet aggregation, similar to wild-type platelets [41]. This discrepancy may be attributed to the fact that the 20 μM in vitro concentration of lyso-SM required to decrease α_IIb_β_3_ integrin activation and platelet aggregation in vitro is significantly higher than the levels observed in the plasma of patients with ASMD. The median plasma concentrations are 0.34 μM for the chronic forms of ASMD and 0.83 μM for the infantile neurovisceral form [41,59]. The mechanism by which lyso-sphingomyelin inhibits α_IIb_β_3_ integrin activation and platelet aggregation remains an open question for future investigation [60].

Blood loss from the wound site is significantly higher in *SMPD1* knockout mice and lower in *SMS2* knockout mice compared to their wild-type counterparts [58]. However, another study reported contradictory results, demonstrating that *SMS2* knockout platelets exhibit a remarkable decrease in aggregation, spreading, clot retraction, and in vivo thrombosis, evaluated via the FeCl_3_ model. Similar detrimental effects on platelet activation were detected in wild-type platelets treated with D609, an inhibitor of the sphingomyelin synthase 2 enzyme [61]. The PLCγ/PI3K/PKB signaling pathway is inhibited in both *SMS2* knockout platelets and D609-treated wild-type platelets [62]. Crucially, FeCl_3_-induced thrombus formation relies heavily on collagen-dependent pathways secondary to endothelial damage, rather than tissue factor-dependent thrombin generation [58,63].

Sphingomyelin is an important constituent of platelet lipid rafts. Fibrin is translocated to these sphingomyelin-rich rafts, which act as platforms where extracellular fibrin and intracellular actomyosin connect to facilitate blood clot retraction [64]. This process might be abnormal in acid sphingomyelinase deficiency, although this has yet to be established.

Interestingly, *SMPD1* knockout platelets demonstrate a protective effect against transfusion-related acute lung injury (TRALI) in a murine model [65]. This phenomenon may be due to the fact that TRALI is exacerbated by activated platelets, and, as previously mentioned, acid sphingomyelinase deficiency significantly decreases the platelet activation level [40,66].

### 2.2. Coagulation Factor Impairment in ASMD

The TF-FVIIa complex plays a significant role in thrombus formation by activating FIX and FX [67,68]. Sphingomyelin appears to inhibit tissue factor (TF) activation, and this effect is not caused by interference with FVIIa or TF-FVIIa complex formation [69]. Sphingomyelin accumulation maintains TF in an encrypted state; conversely, ASMase increases ATP-induced TF decryption and activation and the release of TF-positive microvesicles from macrophages, whereas inhibition of ASMase hinders these processes [70]. Inhibition of ASMase also weakens LPS- and LPS+ATP-induced procoagulant activity of TF, thereby reducing thrombin generation in murine models [71].

Knockout of the *SMPD1, SMPD3* and *SMPD4* genes, which encode the enzymes ASMase, nSMase 2, and nSMase 3, respectively, causes an elevation of sphingomyelin in the plasma membrane and inhibits ATP-induced activity of tissue factor in endothelial cells and macrophages. Similarly, fibroblasts from patients with ASMase deficiency and elevated sphingomyelin levels exhibit decreased tissue factor activity compared to wild-type fibroblasts [58].

**Figure 2:**
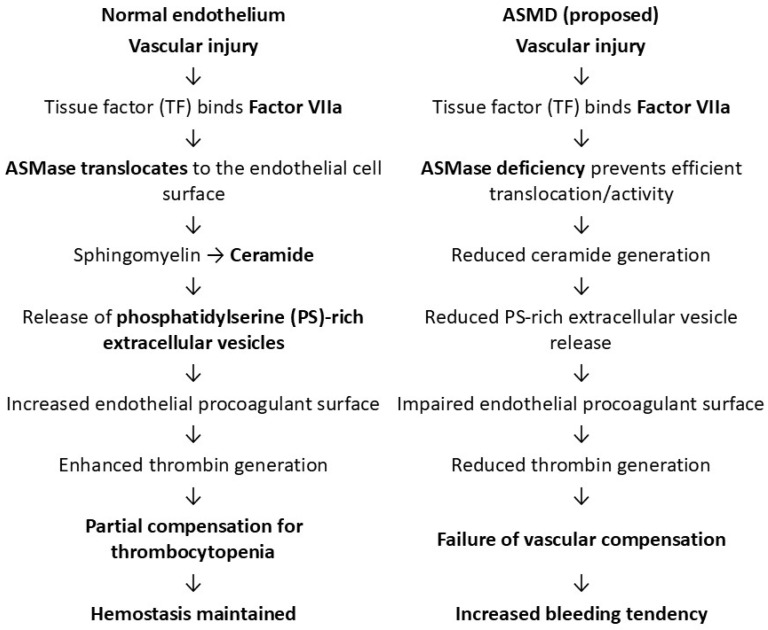
Schematic illustration of the normal compensatory mechanism following vascular injury (**left**) and the proposed mechanism underlying the failure of endothelial vesicle-mediated compensation in ASMD (**right**).

There is another mechanism that may render ASMD patients more vulnerable to thrombocytopenia-related bleeding compared to patients with other thrombocytopenic disorders (Figure 2). Phosphatidylserine-rich extracellular vesicles released from endothelial cells in response to FVIIa treatment exhibit elevated procoagulant activity, and these particles could potentially counteract platelet deficiency [72]. Factor VIIa mediates the relocalization of active ASMase to the endothelial cell surface, which subsequently triggers the release of extracellular vesicles through ceramide formation [73]. This compensatory mechanism is impaired or completely absent in *SMPD1* knockout models [74]. Thus, the disruption of this pathway due to ASMase deficiency may exacerbate the bleeding tendency already present due to platelet activation defects and thrombocytopenia levels in patients with ASMD.

Considering that liver failure and severe hepatic dysfunction are hallmark features of ASMD [24], these conditions may impede the synthesis of coagulation factors, potentially resulting in secondary coagulopathy [75].

### 2.3. Role of Acid Sphingomyelinase in Platelet-Mediated Tumor Metastasis

ASMase inhibition blocks the buildup of ceramide in aging murine platelets [65]. Ceramide is classically associated with apoptosis and growth suppression; however, accumulating evidence implies that this association is not absolute [76]. There is significant evidence that platelets can act as tumor promoters via various mechanisms [77,78]. In murine models, platelet-secreted acid sphingomyelinase has been shown to promote hematogenous metastasis, particularly in melanoma. Acid sphingomyelinase secretion by activated platelets triggers ceramide formation, inducing the clustering and activation of α5β1 integrins on the surface of melanoma cells, which ultimately promotes tumor cell adhesion [79]. The involvement of ASMase and platelets in melanoma metastasis is further supported by the use of the pharmacological ASMase inhibitor, ARC39, which attenuates metastasis formation [80].

Initially, it was proposed that acid sphingomyelinase activates p38 MAP kinase in melanoma cells, thereby triggering β1 integrin stimulation, which in turn enhances hematogenous melanoma metastasis [81]. However, subsequent evidence revealed a complex, bidirectional loop in which acid sphingomyelinase secretion is, conversely, activated by p38 MAPK [82]. *SELP* knockout murine platelets do not promote metastasis; thus, P-selectin appears necessary for this process. The interaction of P-selectin with its ligands, such as PSGL-1, activates the p38 MAPK pathway, leading to ASMase secretion and subsequent tumor metastasis [82].

Although these experimental studies demonstrate an important role for platelet-derived acid sphingomyelinase in melanoma metastasis, their clinical relevance to ASMD remains uncertain. To date, no studies have demonstrated altered melanoma incidence or metastasis in patients with ASMD. Moreover, ASMD has been associated with an increased overall cancer prevalence, indicating that these experimental findings should not be interpreted as evidence of a protective effect in patients [83]. Furthermore, this mechanism does not explain the bleeding tendency or qualitative platelet abnormalities observed in ASMD.

### 2.4. Platelet Lipidome Changes in ASMD Models

Lipidomic studies show that *SMPD1* knockout murine platelets exhibit significantly higher lyso-SM levels compared to wild-type controls. The *SMPD1* knockout platelets also exhibit lower levels of sphinganine and sphingosine, reflecting a disruption of the Golgi apparatus, which serves as the primary site of synthesis for these lipids. This dysfunction likely results in impaired biogenesis and transport of platelet granules, since the trans-Golgi network is required for granule maturation in megakaryocytes [84,85].

Interestingly, ceramide with the sphingoid base/long-chain base d18:2/24:1 is significantly decreased, while the levels of d18:1/24:1, d18:0/24:1 ceramides, and sphingomyelin do not differ in *SMPD1* knockout murine platelets. This identical lipidomic phenotype is triggered in wild-type platelets by the addition of lyso-SM, which leads to impaired CRP-mediated activation, as previously described [41]. Further studies are required to elucidate the link between these lipidomic alterations and the observed defects in platelet activation associated with ASMD.

Given that ASMase mediates a crucial step in platelet S1P generation [54], and S1P is an important signaling molecule that is required for thrombus formation and stabilization at vascular injury sites [86], a decrease in S1P represents a key potential pathogenetic mechanism of the impaired hemostasis observed in patients with ASMD.

Platelet-activating factors (PAFs) are a family of glycerophosphocholines capable of inducing platelet aggregation through the G-protein-coupled PAF receptor (PAFR) [87,88,89]. In vivo, two PAF species are present: the potent PAFR activator alkyl-PAF and its much less biologically active acylated analog [89]. PAFs are secreted by various cell types, including platelets, macrophages, monocytes, neutrophils, eosinophils, endothelial cells, fibroblasts, and cardiomyocytes [90,91]. It is well-established that PAF antagonism can inhibit thrombosis [92].

**Figure 3:**
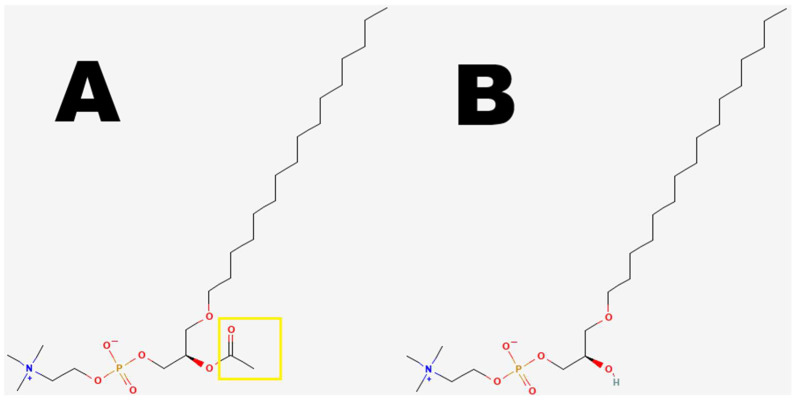
Chemical structures of PAF (**A**) and lyso-PAF (**B**), with the deacetylated acetyl group highlighted in a yellow square. The 2D structure image of platelet-activating factor was obtained from the National Center for Biotechnology Information PubChem Compound Summary (CID 108156), available at: https://pubchem.ncbi.nlm.nih.gov/compound/Platelet-activating-factor, accessed on 29 June 2026. The image is licensed under a CC0 1.0 Universal Public Domain Dedication, available at https://creativecommons.org/publicdomain/zero/1.0/deed.en (accessed on 29 June 2026).

Lyso-forms of PAFs are secondary storage products observed in various lysosomal storage diseases, including infantile neurovisceral ASMD and Niemann–Pick disease type C (Figure 3) [93,94]. Although lyso-derivatives of PAFs are commonly thought to be biologically inactive molecules, emerging data suggest that they exhibit several proinflammatory and prooncogenic properties; however, these effects might be attributed to contamination with or reacetylation to PAF [89,95,96,97,98]. Lyso-PAFs can be derived from the hydrolysis of PAFs and plasma membrane alkylacylphosphatidylcholine or transacylation between alkylacylphosphatidylcholine and lysoplasmalogen [93,97].

In both ASMase-deficient patients and *SMPD1* knockout mice, lyso-PAF levels in brain tissue significantly correlate with lyso-sphingomyelin, a marker of ASMD [59,93]. Meanwhile, PAF levels are not elevated in animal models of Niemann–Pick disease type C [94]. Lyso-PAF does not trigger aggregation in human platelets in vitro; instead, it inhibits thrombin-induced platelet aggregation via cAMP generation and activation of protein kinase A (PKA) [99,100]. Thus, lyso-PAF-mediated inhibition of platelet aggregation may also play a role in the hemostatic abnormalities observed in patients with ASMD. Pretreatment with lyso-PAF fails to prevent PAFR activation by alkyl-PAF. Interestingly, in murine models, pretreatment with lyso-PAF significantly reduces the lethality of alkyl-PAF [100].

Although a significant correlation between lyso-PAF and lyso-sphingomyelin levels has been established in murine brain tissues, it remains unknown whether lyso-PAF accumulates in the plasma or platelets of ASMD patients. Consequently, while the in vitro inhibitory effects of lyso-PAF on human platelet aggregation suggest a potential bleeding mechanism, this pathway remains speculative in the context of clinical ASMD and requires direct lipidomic validation in patient samples.

### 2.5. Platelet Desialylation as a Possible Mechanism of Thrombocytopenia in ASMD

The removal of surface sialic acid, also known as desialylation, is a crucial mechanism of platelet clearance in the liver [101]. As platelets age or become activated in the circulation, they secrete endogenous neuraminidases that cleave terminal sialic acid residues of surface glycoproteins and glycolipids, exposing galactose residues. These exposed carbohydrates are then recognized by specific lectin-like receptors, such as the Ashwell–Morell receptor on hepatocytes and Kupffer cells, driving hepatic clearance [102]. Desialylation is a key mechanism of pathologically elevated platelet clearance in patients with immune thrombocytopenia [103]. Similarly, platelets from patients with glycosylation disorders stemming from mutations in the *GNE* and *SLC35A1* genes exhibit both defective sialylation and thrombocytopenia [42,43].

Since sialic acid metabolism is closely intertwined with sphingolipid metabolism [104,105], elevated desialylation represents an attractive hypothesis for platelet clearance in ASMD, mirroring mechanisms observed in other metabolic and immune disorders [106,107]. However, it must be explicitly emphasized that no studies on platelet desialylation have been conducted in either ASMD patients or animal models to date. Consequently, the involvement of this mechanism in ASMD-associated thrombocytopenia remains theoretical and warrants further investigation.

### 2.6. Disruption of Acidic Compartment Ca^2+^ Flux in a Related Disorder

Evaluating platelet pathology in Niemann–Pick disease type C (NPC)—a related disorder characterized by sphingomyelin accumulation but caused by mutations in a different gene—provides valuable insights. The NPC1 protein is expressed in platelets and megakaryocytes. Similar to observations in ASMD, P-selectin expression and ATP secretion are both decreased in NPC, as confirmed by electron microscopy; however, unlike in ASMD, platelet aggregation is also diminished. In vitro differentiated megakaryocytes from NPC1 patients exhibit hyperproliferation of immature cells, altered CD63+ granules, and abnormal intracellular cholesterol accumulation. Additionally, NPC1-depleted zebrafish embryos exhibit thrombocytopenia and mild anemia [108]. Conversely, murine models demonstrate increased platelet counts, defective platelet aggregation and prolonged bleeding times. Electron microscopy reveals abnormal ultrastructure in these murine platelets, mirroring the defects observed in the megakaryoblastic cell line MEG-01 treated with a pharmacological NPC1 inhibitor, which displays impaired lipid storage and acidic compartment Ca^2+^ flux. Concurrently, clinical data indicate that NPC1 patients exhibit platelet counts at the lower end of the normal range and platelet volumes clustered at the higher end, with some patients exhibiting mild macrothrombocytopenia [109,110,111,112]. Finally, *NPC1* knockout murine platelets show elevated sphingosine levels and localized defects in NAADP-dependent and SERCA3-dependent Ca^2+^ flux from acidic cellular compartments, such as lysosomes and lysosome-related organelles [113], compared to wild-type controls [44].

While Ca^2+^ flux from acidic compartments has been demonstrated to be defective in NPC1 knockout murine platelets, this parameter remains completely unstudied in ASMase-deficient platelets. Given that sphingomyelin accumulation occurs in both disorders, NPC1 data provide a compelling rationale to investigate similar defects in ASMD. Nevertheless, crucial phenotypic differences exist—such as impaired platelet aggregation in NPC1 models versus preserved aggregation in SMPD1-knockout mice—underscoring the risk of direct extrapolation. Calcium signaling disturbances in ASMD must therefore be treated as a tentative pathogenetic link until direct experimental evidence is obtained.

## 3. Conclusions

**Figure 4:**
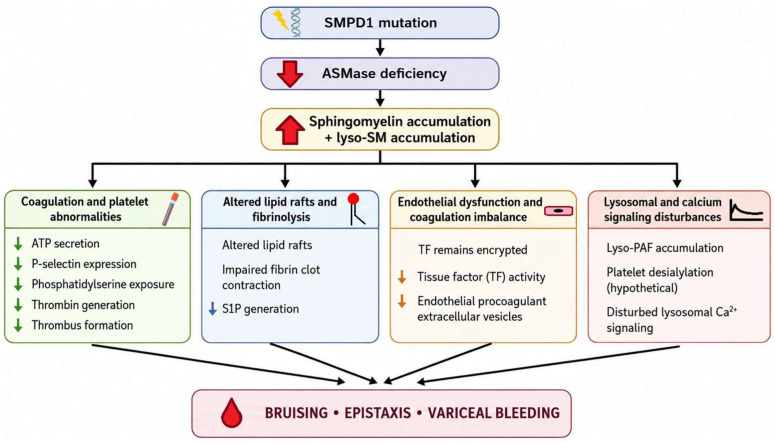
Comprehensive summary of platelet function defects and other hemostatic abnormalities observed in acid sphingomyelinase deficiency. Downward arrows (↓) indicate down-regulation, decreased secretion, or reduction of the respective processes or factors. Red upward arrow icon indicates the accumulation of the respective factors. Abbreviations: ASMase, acid sphingomyelinase; lyso-SM, lysosphingomyelin; S1P, sphingosine-1-phosphate; lyso-PAF, lyso-platelet-activating factor; Ca^2+^, calcium. The DNA, blood tube and lipid icons in the figure were obtained from BioIcons (2026) (image names “DNA_double_helix” by James-Lloyd, “blood_sample” by Marcel Tisch and “Phospholipid” by Cléber-Gomes), all licensed under CC0 1.0 Universal Public Domain Dedication https://creativecommons.org/publicdomain/zero/1.0/deed.en. https://bioicons.com/ (accessed on 25 June 2026). Graphic icons are used for illustrative purposes only.

In summary, it can be stated that ASMD is a complex multisystemic disease, in which hemostatic abnormalities go beyond secondary thrombocytopenia (Figure 4). Although sphingomyelin accumulation is classically associated with visceral and neurological symptoms, emerging data demonstrate the presence of various hemostatic defects. Although systematic platelet-function studies in patients with ASMD are currently lacking, biological models reveal intrinsic platelet defects.

Several limitations of the experimental models used in the described research warrant discussion. First, while the *SMPD1* knockout mouse model is a valuable tool for studying ASMD, it represents a complete loss of function. This phenotype does not fully capture the highly variable residual enzyme activity and the broad genotype–phenotype spectrum observed in human patients. Partial inhibition models, such as gene knockdown, may provide a more representative alternative to complete knockouts. Consequently, the translational applicability of murine findings to clinical ASMD requires caution, given that murine metabolism differs significantly from that of humans. Second, pharmacological models utilizing functional inhibitors of acid sphingomyelinase (FIASMAs), such as amitriptyline and fluoxetine, serve as an acute disruption rather than a genetic equivalent to ASMD. Since FIASMAs are multi-target molecules, they are also known to exert ASMase-independent side effects on platelets, including the modulation of various surface receptors and intracellular signaling pathways, which limits their utility as true ASMD models. Therefore, acute pharmacological inhibition, absolute genetic deficiency, and chronic lysosomal sphingomyelin accumulation represent distinct pathophysiological states and should not be treated as interchangeable conditions. Future studies are needed to delineate the precise contributions of chronic lipid accumulation versus acute enzymatic suppression on platelet function.

Regarding platelet function, ASMase deficiency causes the attenuation of ATP secretion, P-selectin expression, and thrombin generation, all of which significantly compromise the capacity for thrombus formation. Changes in membrane lipid composition, mediated by sphingomyelin and cholesterol accumulation, may impair platelet adhesion and aggregation. The increased risk of bruising and bleeding is likely to be caused by a combination of thrombocytopenia, intrinsic platelet defects, and the impairment of compensatory endothelial hemostasis mechanisms. However, a clear distinction must be maintained between verified pathological features and proposed mechanisms. An inverse correlation between spleen volume and platelet count is well-established in patients, reflecting splenic sequestration secondary to splenomegaly, although the resulting thrombocytopenia is insufficient to fully account for the observed bleeding events. While secondary thrombocytopenia and impaired thrombin generation are documented in biological models of ASMD, the roles of platelet desialylation, the inhibition of thrombin-induced platelet aggregation by secondary lyso-PAF accumulation, and lysosomal Ca^2+^ signaling defects are inferred only from related disorders or indirect systems, remaining to be experimentally proven in ASMD.

In conclusion, establishing definitive links between sphingomyelin accumulation and platelet dysfunction remains a critical priority for optimizing ASMD management. Future research should prioritize prospective cohort studies in ASMD patients to evaluate comprehensive platelet parameters, including aggregation, ATP secretion, flow cytometric activation markers, phosphatidylserine exposure, thrombin generation, and granule morphology. Notably, tracking these parameters before and after olipudase alfa therapy would provide crucial insights into the impact of enzyme replacement therapy on hemostasis. Correlating these functional changes with platelet counts, splenic volume, liver disease severity, lyso-sphingomyelin levels, genotype, and prospective bleeding events will enhance bleeding risk stratification. Furthermore, the precise molecular mechanisms underlying the multitargeted inhibition of platelet activation by lyso-sphingomyelin require characterization. Further study is also warranted to evaluate the degree of intracellular Ca^2+^ signaling impairment—paralleling findings in Niemann–Pick disease type C—potentially opening new therapeutic possibilities for correcting hemostatic abnormalities in this population. Understanding these mechanisms is essential to improve clinical management and minimize the risk of life-threatening hemorrhages in ASMD.

## Figures and Tables

**Table 1:** Overview of the main clinical forms of ASMD.

Form	Severity	Life Expectancy	Key Manifestations
Infantile neurovisceral	Significant [16,17]	Significantly reduced, high mortality in early infancy [16]	Rapid CNS deterioration [16]
Chronic visceral	Mild to moderate [18]	Survive into adulthood [18,21,22]	Hepatosplenomegaly, visceral symptoms [18]
Chronic neurovisceral	Moderate to significant [17,19,20]	Survival into adulthood rare but possible [19,20,21,22]	Slower CNS deterioration and visceral symptoms [19,20]

## Data Availability

No new data were created or analyzed in this study.

## Abbreviations

The following abbreviations are used in this manuscript:

ASMD	Acid sphingomyelinase deficiency
S1P	Sphingosine-1-phosphate
CNS	Central nervous system
ADP	Adenosine diphosphate
ASMase	Acid sphingomyelinase
PKC	Protein kinase C
CRP	Collagen-related peptide
ATP	Adenosine triphosphate
FIASMA	Functional acid sphingomyelinase inhibitor
Lyso-SM	Lyso-sphingomyelin
PLCγ	Phospholipase C gamma
PI3K	Phosphoinositide 3-kinase
PKB	Protein kinase B
D609	Tricyclodecan-9-yl-xanthogenate
TRALI	Transfusion-Related Acute Lung Injury
TF	Tissue factor
FVIIa	Factor VIIa
FIX	Factor IX
FX	Factor X
LPS	Lipopolysaccharide
nSMase	Neutral sphingomyelinase
ARC39	1-aminodecylidene bis-phosphonic acid
MAPK	Mitogen-activated protein kinase
PSGL-1	P-selectin glycoprotein ligand 1
PAF	Platelet-activating factor
PAFR	Platelet-activating factor receptor
Alkyl-PAF	Alkyl-platelet-activating factor
Lyso-PAF	Lyso-platelet-activating factor
CID	Compound identification
cAMP	Cyclic adenosine monophosphate
PKA	Protein kinase A
NPC1	Niemann–Pick disease, type C1 (protein)
CD	Cluster of differentiation
MEG-01	Megakaryoblastic leukemia cell line-01
NAADP	Nicotinic acid adenine dinucleotide phosphate
SERCA3	Sarcoplasmic/endoplasmic reticulum calcium ATPase 3
SM	Sphingomyelin
